# The Tick Cell Biobank: A global resource for *in vitro* research on ticks, other arthropods and the pathogens they transmit

**DOI:** 10.1016/j.ttbdis.2018.05.015

**Published:** 2018-07

**Authors:** Lesley Bell-Sakyi, Alistair Darby, Matthew Baylis, Benjamin L. Makepeace

**Affiliations:** a Institute of Infection and Global Health, University of Liverpool, Liverpool Science Park IC2, 146 Brownlow Hill, Liverpool L3 5RF, United Kingdom; b Institute of Integrative Biology, University of Liverpool, Biosciences Building, Crown Street, Liverpool L69 7ZB, United Kingdom; c NIHR Health Protection Research Institute in Emerging and Zoonotic Infections, Institute of Infection and Global Health, University of Liverpool, The Ronald Ross Building, 8 West Derby Street, Liverpool L69 7BE, United Kingdom

**Keywords:** Tick cell line, Arthropod, Mite, Midge, Sand fly, Intracellular bacteria

## Abstract

Tick cell lines are increasingly used in many fields of tick and tick-borne disease research. The Tick Cell Biobank was established in 2009 to facilitate the development and uptake of these unique and valuable resources. As well as serving as a repository for existing and new ixodid and argasid tick cell lines, the Tick Cell Biobank supplies cell lines and training in their maintenance to scientists worldwide and generates novel cultures from tick species not already represented in the collection. Now part of the Institute of Infection and Global Health at the University of Liverpool, the Tick Cell Biobank has embarked on a new phase of activity particularly targeted at research on problems caused by ticks, other arthropods and the diseases they transmit in less-developed, lower- and middle-income countries. We are carrying out genotypic and phenotypic characterisation of selected cell lines derived from tropical tick species. We continue to expand the culture collection, currently comprising 63 cell lines derived from 18 ixodid and argasid tick species and one each from the sand fly *Lutzomyia longipalpis* and the biting midge *Culicoides sonorensis*, and are actively engaging with collaborators to obtain starting material for primary cell cultures from other midge species, mites, tsetse flies and bees. Outposts of the Tick Cell Biobank will be set up in Malaysia, Kenya and Brazil to facilitate uptake and exploitation of cell lines and associated training by scientists in these and neighbouring countries. Thus the Tick Cell Biobank will continue to underpin many areas of global research into biology and control of ticks, other arthropods and vector-borne viral, bacterial and protozoan pathogens.

## 1 Introduction

While ticks are economically devastating ectoparasites and the most important arthropod vectors of livestock diseases, and second only to mosquitoes as vectors of human pathogens, they traditionally lagged behind insects in the field of cell line establishment (Pudney, 1987). In recent years, this deficit has been greatly reduced: for example, the online resource Cellosaurus https://web.expasy.org/cellosaurus/ lists 80 mosquito cell lines, of which less than half have been used in studies published since 2000, and 84 tick cell lines, of which at least 66 are still extant and almost all have been used in published studies in the past 9 years.

Most tick cell lines were derived from embryos (Bell-Sakyi et al., 2007), as tick eggs are the most easily-handled developmental stage and a plentiful source of relatively-undifferentiated starting material with the potential, in theory at least, of rapid growth. In brief, engorged female ticks are surface-sterilised before oviposition commences, egg batches are surface-sterilised again when the developing embryos are visible, the eggshells are crushed gently in balanced salt solution or culture medium to release the embryos and the resultant tissue suspension is incubated in sealed containers at between 28 and 34 °C (for example, Bell-Sakyi, 1991; Bell-Sakyi et al., 2009; Mattila et al., 2007; Munderloh et al., 1994; Yunker et al., 1981). Medium is changed weekly on average; the most widely-used culture media are based on L-15 (Leibovitz) medium supplemented with tryptose phosphate broth and foetal bovine serum, and the modified L–15 B medium of Munderloh and Kurtti (1989) has proved to be very useful. Significant cell growth may commence in a proportion of primary cell cultures between 8 weeks and 3 years later, depending on the tick species, and a proportion of these cultures may eventually yield cell lines after 1–7 years *in vitro*. Cell lines have also been established from moulting larval (developing nymph) *Amblyomma variegatum* and moulting nymphal (developing adult) *Rhipicephalus appendiculatus* and *Rhipicephalus evertsi*. Specific tick organs such as midgut, salivary glands, haemocytes or ovaries have not, as yet, yielded cell lines despite numerous attempts to maintain and propagate them *in vitro* (Fujisaki et al., 1975; Hoffmann et al., 1970; Mosqueda et al., 2008; Rehacek, 1976; Rezende et al., 2015; Bell-Sakyi unpublished observations).

Tick cell lines, regardless of the species and laboratory of origin, share a number of characteristics (Bell-Sakyi et al., 2007), some of which underline their dissimilarity to vertebrate and insect cell lines. In particular, all are both genotypically and phenotypically heterogeneous, having been derived from multiple individuals; most do not require regular subculture and can be maintained as individual cultures with weekly medium change for many months or years (maximum 17 years at the time of writing); all are relatively slow-growing, tolerate high densities and grow in three dimensions; although cryopreservation can be challenging, ixodid tick cells frozen and thawed rapidly can have good viability after many years in liquid nitrogen (maximum 36 years at the time of writing). In contrast, cell lines derived from the argasid tick species *Carios capensis* (Mattila et al., 2007) and *Ornithodoros moubata* (Bell-Sakyi et al., 2009) comprise cells which are relatively fragile and difficult or impossible to cryopreserve.

## 2 Tick cell lines as research tools

**Fig. 1:**
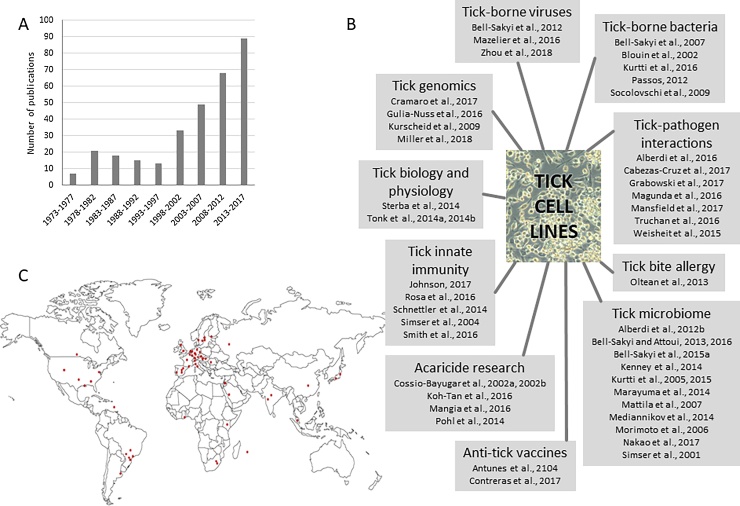
**Impact of tick cell lines and the Tick Cell Biobank in global tick and tick-borne disease research. A.** Number of publications reporting generation and/or use of tick cell lines over the past 45 years. Data from https://www.liverpool.ac.uk/infection-and-global-health/research/tick-cell-biobank/bibliography/. **B.** Selected reviews and original research papers illustrating the broad spectrum of research topics in which tick cell lines play a role: Antunes et al. (2014), Bell-Sakyi and Attoui (2013), Bell-Sakyi and Attoui (2016), Blouin et al. (2002), Cabezas-Cruz et al. (2017), Contreras et al. (2017), Cossio-Bayugar et al. (2002a), Cossio-Bayugar et al. (2002b), de Abreu et al. (2013), Grabowski et al. (2017), Gulia-Nuss et al. (2016), Hoffmann et al. (1970), Johnson (2017), Kenney et al. (2014), Kurscheid et al. (2009), Kurtti et al. (2015), Kurtti et al. (2008), Magunda et al. (2016), Mansfield et al. (2017), Marayuma et al. (2014), Mattila et al. (2006), Mediannikov et al. (2014), Morimoto et al. (2006), Nakao et al. (2017), Oltean et al. (2013), Passos (2012), Rosa et al. (2016), Schnettler et al. (2014), Simser et al. (2004), Simser et al. (2001), Smith et al. (2016), Socolovschi et al. (2009), Sterba et al. (2014), Tonk et al. (2014a), Tonk et al. (2014b) and Truchan et al. (2016). **C.** Locations of institutes working with tick cell lines supplied by the Tick Cell Biobank (due to the small scale, it is not possible to indicate all the institutes in UK and Europe individually).

Tick cell lines are becoming increasingly important laboratory tools (Bell-Sakyi et al., 2007), facilitating research on many aspects of biology, physiology and control of ticks and tick-borne pathogens, exemplified by the rapid increase in publications reporting their use since the turn of the century (Fig. 1A). This review cannot undertake to cite all recent relevant publications, but draws attention to reviews and selected original research articles of note in their particular field, as listed in Fig. 1B. Since the first tick cell lines were established nearly 50 years ago, they have been applied extensively to isolate, propagate and study tick-borne viruses and bacteria, and in numerous studies on tick-pathogen interactions. Tick cell lines have also been widely used in studies on tick genomics, tick biology and physiology, innate immunity, tick bite allergy, the tick microbiome, anti-tick vaccines, mode-of-action of acaricides and development of acaricide resistance (Fig. 1B).

Some recent studies particularly highlight the essential role of tick cell lines as research tools, and the advantages and opportunities that they provide for virologists, bacteriologists and tick biologists. Using an *Ixodes ricinus*-derived cell line and the model tick-borne phlebovirus Uukuniemi virus, Mazelier et al. (2016) showed that tick cell-derived virus particles have specific molecular and structural properties that enhance their infectivity for mammalian cells. Moreover, by demonstrating the existence of flavivirus RNA and protein inside exosomes produced by Langat virus-infected *Ixodes scapularis* cells, and successfully using the exosome fraction to infect human cells, Zhou et al. (2018) provided evidence indicating that tick-borne viruses may use tick cell-derived exosomes as a vehicle for transmission to the vertebrate host. Apathogenic rickettsial endosymbionts of ticks can be used as laboratory models for highly pathogenic *Rickettsia* species; two such endosymbionts, *Rickettsia peacockii* and *Rickettsia buchneri*, isolated using tick cell lines (Kurtti et al., 2005, Kurtti et al., 2015), have been transformed to express fluorescent proteins, thereby enhancing their application for *in vitro* and *in vivo* study (Kurtti et al., 2016). RNA sequencing of *I. ricinus* and *I. scapularis* cell lines infected with a human isolate of *Anaplasma phagocytophilum* revealed cell line-specific responses that mirrored those seen *in vivo* in different tick tissues, respectively midgut and haemocytes (Alberdi et al., 2016). Tick cell lines are increasingly being used as a cheaper and more ethical alternative to live ticks in some aspects of acaricide research; use of acaricide-treated tick cells or cell lines derived from acaricide-resistant ticks has thrown light on the role of ABC transporters (ABCt) in acaricide resistance. Pohl et al. (2014) derived an ivermectin-resistant subline of a *Rhipicephalus microplus* cell line and demonstrated increased levels of ABCt genes in the resistant cells; in contrast, Mangia et al. (2016) did not detect significant effects of ivermectin treatment on ABCt genes in an *I. ricinus* cell line, despite the concentration of ivermectin being twice that used by Pohl et al. (2014). Comparing cell lines derived from acaricide-susceptible and acaricide-resistant *R. microplus*, Koh-Tan et al. (2016) demonstrated significantly higher expression of an ABCt gene and identified a novel β-adrenergic octopamine receptor gene in the resistant cell line.

Several studies have proved the potential for genetic manipulation of multiple tick cell lines through transient and stable transfection (Barry et al., 2013; Kurtti et al., 2006, Kurtti et al., 2008; Machado-Ferreira et al., 2015; Pedra et al., 2010) and development of a reporter gene system (Tuckow and Temeyer 2015).

In contrast, few studies have tackled the difficult topic of characterisation of the multiple phenotypes present in tick cell lines (de Abreu et al., 2013; Esteves et al., 2008; Oliver et al., 2015) and have focused on a handful of cell lines derived from *I. scapularis* and *R. microplus*. Transcriptome datasets available for cell lines derived from six mostly North American tick species (*Amblyomma americanum*, *Dermacentor andersoni*, *Dermacentor variabilis*, *I. ricinus*, *I. scapularis* and *Rhipicephalus sanguineus* sensu lato) at https://www.ncbi.nlm.nih.gov/bioproject/238805 and transcriptomic and proteomic datasets generated in tick-pathogen interaction studies (Alberdi et al., 2016; Grabowski et al., 2016; Villar et al., 2015; Weisheit et al., 2015) can be exploited in whole cell line characterisation, but do not differentiate between individual cell phenotypes present in those cell lines. So far, all attempts to clone tick cell lines have been unsuccessful (Munderloh et al., 1994; Bell-Sakyi, unpublished results); until a suitable technique is developed, researchers must deal with the reality of tick cell line heterogeneity, which can be advantageous for some applications such as isolation of microorganisms (Bell-Sakyi et al., 2012).

## 3 The Tick Cell Biobank

The Tick Cell Biobank was founded in 2009 as a collaboration between the Universities of Edinburgh (the host institute), Minnesota and Texas A&M, with 3 years’ funding from the Wellcome Trust, to create a repository for existing tick cell lines, a resource to support their successful dissemination worldwide and a facility for establishment of new tick cell lines. At that time we housed 47 continuous tick cell lines originally established in five laboratories (the three founders mentioned above plus London School of Hygiene and Tropical Medicine and Rocky Mountain Laboratories) of which 36 were sufficiently robust to be distributed. From the start, an important part of our remit was to provide training in tick cell line maintenance in order to facilitate successful transfer to, and application in, recipient laboratories; latterly this was extended to include training in primary tick cell and organ culture and cell line establishment.

In 2012 the Tick Cell Biobank moved to The Pirbright Institute and thence in 2017 to the University of Liverpool https://www.liverpool.ac.uk/infection-and-global-health/research/tick-cell-biobank/, by which time the numbers of tick cell lines available for distribution had increased to 46 (Table 1). The total number of tick cell lines housed in the Tick Cell Biobank now stands at 63, including new unpublished cell lines derived from the ixodid ticks *Hyalomma dromedarii*, *R. evertsi*, *R. appendiculatus* and *R. microplus* and the argasid species *Argas reflexus* (Table 2). Primary cultures and early subcultures from the European ixodid species *Dermacentor marginatus, Hyalomma lusitanicum*, *Hyalomma scupense* and *Rhipicephalus bursa* show potential for cell line establishment.

**Table 1:** **Tick cell lines currently available from the Tick Cell Biobank.** Additional cell lines are held in the collection and may be made available on request. The original references for all cell lines are cited in Alberdi et al. (2012b) and/or Bell-Sakyi et al. (2015b), except where indicated below.

Tick species	Geographical origin	Cell line
*Amblyomma americanum*	USA	AAE2, AAE12
*Amblyomma variegatum*	Southern Africa	AVL/CTVM13, AVL/CTVM17
*Carios capensis*	USA	CCE1, CCE2
*Dermacentor albipictus*	USA	DALBE3
*Dermacentor andersoni*	USA	DAE15, DAE100T
*Dermacentor nitens*	USA	ANE58
*Dermacentor variabilis*	USA	DVE1
*Hyalomma anatolicum*	India	HAE/CTVM8, HAE/CTVM9
*Ixodes ricinus*	UK	IRE/CTVM19, IRE/CTVM20
	Germany	IRE11
*Ixodes scapularis*	USA	IDE2, IDE8, ISE6, ISE18
*Ornithodoros moubata*	East Africa	OME/CTVM21, OME/CTVM22, OME/CTVM24, OME/CTVM27
*Rhipicephalus appendiculatus*	East Africa	RA243, RA257
	Kenya	RAE25, RAE/CTVM1, RAN/CTVM3
*Rhipicephalus decoloratus*	Kenya	BDE/CTVM14, BDE/CTVM16
*Rhipicephalus evertsi*	South Africa	REE/CTVM28, REE/CTVM29, REE/CTVM31, REN/CTVM32
*Rhipicephalus microplus*	Mexico	BmVIII-SCC
	Costa Rica	BME/CTVM2, BME/CTVM4
	Colombia	BME/CTVM5, BME/CTVM6
	Mozambique	BME/CTVM23, BME/CTVM30
*Rhipicephalus sanguineus* sensu lato	USA	RSE8, RML-RSE, RML−15*
	France	RSE/PILS35**

* This cell line was deposited in the Tick Cell Biobank as *Dermacentor variabilis* embryo-derived cell line RML-15 (Yunker et al., 1981) at passage 5 but, as previously reported for the cell line now designated RML-RSE (Bell-Sakyi et al., 2015b), was found to be derived from *Rhipicephalus sanguineus* sensu lato by 16S rRNA gene sequencing (Black and Piesman, 1994). Although the sequences of the 456 kDa fragments amplified from the two cell lines are identical (Ana Palomar, personal communication), the lines differ morphologically and RML-RSE was deposited at passage 64, so it has not been possible to determine if the two cell lines are of the same or different origin and the name RML-15 is retained here for the lower passage cell line.

** Koh-Tan et al., 2016.

**Table 2:** **New cell lines derived from ticks and other arthropods currently held in the Tick Cell Biobank (TCB).** All are currently grown at 28 °C.

Arthropod species (geographical origin)	Instar of origin	Cell line name	Culture medium	Passage level at time of writing (year of origin)	Acknowledgements
*Hyalomma dromedarii* (East Africa)	Embryo	HDE/PIPA33	L-15	13 (2010)	Primary culture initiated and maintained for 2 years by Pilar Alberdi; parent tick provided by Timothy Connelley, University of Edinburgh; tick strain originated from ILRI*.
*Hyalomma dromedarii* (East Africa)	Embryo	HDE/PILS37	L-15/H-Lac	12 (2010)	Parent tick provided by Timothy Connelley, University of Edinburgh; tick strain originated from ILRI.
*Hyalomma dromedarii* (East Africa)	Embryo	HDE/LURF39	L-15	6 (2010)	Primary culture initiated and maintained for 7 years by Rennos Fragkoudis; parent tick provided by Timothy Connelley, University of Edinburgh; tick strain originated from ILRI.
*Rhipicephalus evertsi* (South Africa)	Nymph	REN/PIPA34	L-15/H-Lac	15 (2010)	Primary culture initiated and maintained for 2 years by Pilar Alberdi; engorged nymphal ticks provided by Ard Nijhof, Utrecht University.
*Rhipicephalus appendiculatus* (Kenya)	Embryo	RAE/PIPM38	L-15/L-15B	5 (2011)	Primary culture initiated and maintained for 1 month by Martin Palus; parent tick provided by Timothy Connelley, University of Edinburgh; tick strain originated from ILRI
*Rhipicephalus microplus* (Brazil)	Embryo	BME/PIBB36	L-15	12 (2013)	Primary culture initiated and maintained for 5 months by Bruna Baeta at UFRRJ**, deposited in the TCB by Bruna Baeta and Adivaldo Fonseca, UFRRJ.
*Argas reflexus* (Germany)	Embryo	ARE/LULS41	L-15	6 (2015)	Parent tick provided by Jan-Hendrik Forth, Friedrich-Loeffler Institute Riems.
*Lutzomyia longipalpis* (Brazil)	Embryo	LLE/LULS40	L-15/H-Lac/L-15B	14 (2015)	Derived from sand fly eggs (*L. longipalpis* Campo Grande strain, pheromone type (*S*)-9-methylgermacrene-B) provided by Gordon Hamilton and Ann Underhill, Keele University.

* International Livestock Research Institute, Kenya; **Federal Rural University of Rio de Janeiro, Seropedica, Brazil.

Over the nine years since its inception, the Tick Cell Biobank has distributed tick cell lines to 75 institutes in 30 countries in Asia, Africa, the Americas and Europe (Fig. 1C) and trained 88 young scientists from 25 countries, thereby contributing to over 80 articles in international peer-reviewed journals. These, and all other publications reporting use of tick cell lines, primary tick cell cultures and tick organ culture systems, are listed in a bibliographical resource on the Tick Cell Biobank website (https://www.liverpool.ac.uk/infection-and-global-health/research/tick-cell-biobank/bibliography/).

The Tick Cell Biobank continues to generate, store and distribute tick cell lines, and disseminate associated expertise to scientists worldwide. Its remit and funding has now been expanded to include genotypic and phenotypic characterisation of tick cell lines. Until very recently, no tick cell line genome had been sequenced, and only one tick cell line had been subjected to genome size estimation: the *I. ricinus* cell line IRE/CTVM19 was found to have a genome approximately 1.4 times larger than that of intact *I. ricinus* ticks (Cramaro et al., 2017), adding support to the observation that, over time, tick cell lines become polyploidal, with a range of chromosome complements quite different from those of the parent ticks (Bell-Sakyi et al., 2007). This issue of polyploidy was also raised by Miller et al. (2018), who have just published a draft genome sequence of the widely-used *I. scapularis* cell line ISE6; depending on the method used, they estimated that the ISE6 genome was between 6% and 9% larger than that of the parent tick. We aim to sequence the genomes of up to ten tick cell lines, with a focus on those most widely used in research on tick-borne livestock pathogens of importance in the Tropics. In parallel, we will carry out phenotypic characterisation of these and additional tick cell lines, utilising transcriptomics, proteomics, identification of cell phenotype markers, light and electron microscopy, and, if possible, cell cloning. We will actively seek collaborators for these studies, as there are many more tick cell lines in regular use worldwide than can be tackled by a single group, and there is a clear need for better understanding of the cell phenotypes present within each cell line and how these affect cell line performance in research on tick-pathogen interactions and tick control.

## 4 Tick-borne bacteria in the Tick Cell Biobank

The Tick Cell Biobank has supported numerous in-house and collaborative studies to isolate, propagate and characterise intracellular tick-borne bacteria. The wide range of tick species, many represented by multiple cell lines, facilitates determination of the most suitable host cells for *in vitro* propagation of particular bacterial species. This is exemplified by, at one end of the spectrum, the extremely fastidious *Anaplasma centrale* that only grew in two out of 32 cell lines tested (Bell-Sakyi et al., 2015b) and, at the other end, the much more catholic *Ehrlichia* spp. that grew in almost all tick cell lines tested (Bell-Sakyi, 2004; Cabezas-Cruz et al., 2016; Ferrolho et al., 2016; L. Bell-Sakyi unpublished observations). Such studies can provide insights into the actual and potential range of possible vector species and the factors governing ability to transmit a particular pathogen, bearing in mind that vector competence is multi-factorial and tick cell lines may not fully represent the phenotypic and functional spectrum of cells and tissues present in the intact tick. The ability of individual tick cell cultures to survive for many months without subculture facilitates isolation of fastidious, slow-growing bacterial species that may be present in the inoculum at extremely low levels. The Tick Cell Biobank houses a small collection of intracellular tick-borne bacteria of the genera *Anaplasma*, *Ehrlichia*, *Rickettsia* and *Spiroplasma*, all of which can be propagated in one or more tick cell lines (Table 3).

**Table 3:** **Tick-borne bacteria held in the Tick Cell Biobank.** All species and strains of intracellular bacteria are stored as aliquots of one or more infected tick cell lines in vapour-phase liquid nitrogen.

Bacterial species (strain)	Host (country of origin)	References
*Anaplasma centrale* (Israeli vaccine strain)	Bovine (South Africa)	Shkap et al. (2008); Bell-Sakyi et al. (2015b)
*Anaplasma phagocytophilum* (Old Sourhope)	Sheep (Scotland)	Foster and Cameron (1970); Woldehiwet et al. (2002)
*Anaplasma phagocytophilum* (Perth)	Sheep (Scotland)	Woldehiwet (2010)
*Anaplasma phagocytophilum* (Harris)	Sheep (Scotland)	Woldehiwet (2010)
*Anaplasma phagocytophilum* (Feral Goat)	Goat (Scotland)	Woldehiwet and Horrocks (2005)
*Anaplasma phagocytophilum* (ZW122-05)	Sheep (England)	Z. Woldehiwet personal communication
*Anaplasma phagocytophilum* (ZW144-05)	Bovine (England)	Z. Woldehiwet personal communication
*Anaplasma phagocytophilum* (L610)	Dog (Germany)	Alberdi et al. (2015)
*Ehrlichia canis* (Spain 105)	Dog (Spain)	Zweygarth et al. (2014), Ferrolho et al. (2016).
*Ehrlichia minasensis* (UFMG-EV^T^)	*Rhipicephalus microplus* engorged female tick (Brazil)	Cabezas-Cruz et al. (2016)
*Ehrlichia ruminantium* (Gardel CTVM) *Ehrlichia ruminantium* (Gardel attenuated)	*Amblyomma variegatum* ticks (Guadeloupe) *In vitro* culture (originally from *A. variegatum* ticks, Guadeloupe)	Uilenberg et al. (1985), Bekker et al. (2005). Martinez (1997)
*Ehrlichia ruminantium* (Welgevonden)	*Amblyomma hebraeum* tick (South Africa)	Du Plessis (1985)
*Ehrlichia ruminantium* (Ball 3)	Unspecified mammalian host (South Africa)	Haig (1952)
*Ehrlichia ruminantium* (Sankat 430)	Sheep (Ghana)	Bell-Sakyi et al. (1997)
*Ehrlichia ruminantium* (Pokoase 417)	Sheep (Ghana)	Bell-Sakyi et al. (1997)
*Ehrlichia ruminantium* (Senegal attenuated)	*In vitro* culture (originally from cattle, Senegal)	Jongejan et al. (1988), Jongejan(1991)
*Rickettsia raoultii* (DRET2)	*Dermacentor reticulatus* tick embryos (The Netherlands)	Alberdi et al. (2012a)
*Rickettsia raoultii* (DMAR8)	*Dermacentor marginatus* tick embryos (Russia)	Palomar et al. (in preparation)*
*Rickettsia raoultii* (Białystok 1–5)	*Dermacentor reticulatus* unfed adult ticks (Poland)	Palomar et al. (in preparation)*
*Spiroplasma* sp. Bratislava 1	*Ixodes ricinus* unfed adult ticks (Slovakia)	Bell-Sakyi et al. (2015a)
*Spiroplasma* sp. Białystok 1	*Dermacentor reticulatus* unfed adult male tick (Poland)	Palomar et al. (in preparation)*
*Spiroplasma* sp. DRET3, DRET6, DRET8	*Dermacentor reticulatus* tick embryos (The Netherlands)	Palomar et al. (in preparation)*
*Spiroplasma* sp. IXRI5-6, IXRI8	*Ixodes ricinus* tick embryos (The Netherlands)	Palomar et al. (in preparation)*
*Spiroplasma* sp. DMAR11	*Dermacentor marginatus* tick embryos (Spain)	Palomar et al. (in preparation)*

* Palomar, A.M., Premchand-Branker, S., Alberdi, P., Belova, O., Moniuszko-Malinowska A., Kahl, O., Bell-Sakyi, L. Isolation of known and potentially pathogenic tick-borne microorganisms from European ixodid ticks using tick cell lines.

## 5 Cell lines from neglected arthropods

Another important aspect of the Tick Cell Biobank’s current remit is to establish, house and distribute cell lines derived from “neglected” arthropod species of importance as pathogen vectors, agricultural pests or pollinators. The Biobank already houses the only widely-used biting midge cell line KC, derived from *Culicoides sonorensis* (Wechsler et al., 1991), and is engaged in generating novel cell lines from additional *Culicoides* species as part of the PALE-Blu project (https://cordis.europa.eu/project/rcn/210491_en.html). Following published protocols (Rey et al., 2000; Tesh and Modi, 1983), we have established a novel sand fly cell line, LLE/LULS40, from embryonic *Lutzomyia longipalpis* (Table 2); the parent *L. longipalpis* belonged to the Campo Grande strain from Brazil, which displays the pheromone type (*S*)-9-methylgermacrene-B (Gonzalez et al., 2017). As and when suitable starting material becomes available through collaborators, we are attempting (or will attempt) to generate primary cell cultures with a view to cell line establishment from mites (*Dermanyssus gallinae*, *Leptotrombidium* spp., *Varroa destructor*), tsetse flies (*Glossina* spp.), UK mosquitoes (*Culex* and *Aedes* spp.), bumble bees (*Bombus* spp.) and honey bees (*Apis mellifera*).

## 6 Tick Cell Biobank Outposts

While ticks and tick-borne diseases occur worldwide, the economic burden they impose falls disproportionately on least-developed and lower-middle-income countries (LMIC) in the tropics and sub-tropics. Direct and indirect losses are incurred through costs of tick and tick-borne disease control (acaricides, treatment of clinical cases, vaccines where these exist), reduced productivity, mortality and time expended on control (Minjauw and McLeod, 2003). Acaricide resistance is an increasing problem worldwide, especially for the ubiquitous tropical cattle tick, *R. microplus* (Abbas et al., 2014). Major tick-borne diseases affecting livestock (ruminants, equidae, and poultry) and humans in the tropics include anaplasmosis, babesiosis, borreliosis, ehrlichiosis, rickettsiosis, theileriosis and emerging viral diseases such as African swine fever, Crimean-Congo haemorrhagic fever and severe-fever-with-thrombocytopaenia syndrome. To reduce the economic burden on smallholder farmers and livestock owners in LMIC, new acaricides and/or alternative tick control regimes, and affordable and effective vaccines and/or drug treatments for tick-borne diseases, are urgently needed.

**Fig. 2:**
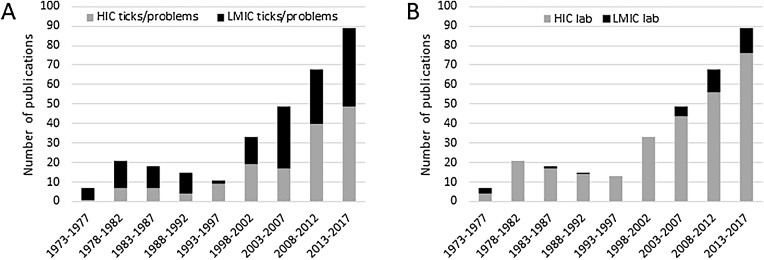
**The disparity between the proportion of tick cell culture research related to LMIC ticks and associated problems and where this research is carried out. A.** Numbers of publications using tick cell culture between 1973 and 2017 relating to high-income countries (HIC, grey bands) and lower- and middle-income countries (LMIC, black bands). **B.** Proportions of the total number of publications that emananted from laboratories in HIC (grey bands) and LMIC (black bands) institutes. Data from https://www.liverpool.ac.uk/infection-and-global-health/research/tick-cell-biobank/bibliography/.

Tick cell lines are increasingly applied in many aspects of research into control of ticks and tick-borne diseases of livestock and humans, particularly those of importance in LMIC. However, most of this research is carried out in high-income countries (HIC) in Europe, North America and the Asia-Pacific region (Japan and Australia). This conundrum is illustrated in Fig. 2: while over half (51%) of the publications utilising tick cell lines since the early 1970s concerned tick and TBD problems in LMIC (Fig. 2A), only 11% of these publications emanated from LMIC institutes (Fig. 2B), most of which are in Brazil. Since the Biobank was established, LMIC countries represent only 8 out of the total 30 countries using TCL from the Biobank; better access to tick cell lines would allow scientists who lack facilities and resources for expensive *in vivo* studies to make a significant contribution to local and global tick-related problems. Currently it is difficult and expensive for LMIC researchers to obtain and maintain existing TCL to support their research, as the cells must be obtained from UK or USA and local training is unavailable. Resources for research on pathogens transmitted by other arthropods such as sand flies, midges, mites and fleas are even more difficult to obtain or non-existent.

To address this deficit, we are establishing outposts of the Tick Cell Biobank in Malaysia, Kenya and Brazil, with the aim of facilitating access to tick cell lines for LMIC scientists, and increasing uptake and application of this technology in their research. The outposts will serve not only their host institute and country, but will disseminate materials and expertise in the surrounding regions of, respectively, South-East Asia, Africa and South America. Each outpost will house a panel of the most popular cell lines, plus some with specific regional relevance, and will distribute cell lines and locally-tailored training in their maintenance. Where possible, they will also attempt to establish novel cell lines from local ticks and other arthropods, building on preliminary studies in Malaysia (Lim et al., 2017) and Brazil (Bruna Baeta and Adivaldo Fonseca, personal communication). We anticipate that the Tick Cell Biobank Outposts should be up and running in early 2020.

## 7 Conclusions and future prospects

Tick cell and tissue culture has progressed a long way since the first short-term cultures carried out in the 1950s and 1960s (reviewed by Yunker, 1987). Continuous cell lines have been established from many of the economically-important tick species prevalent in Europe, Africa and the Americas, and new young scientists in several LMIC have proven their ability to initiate primary cell cultures with a view to establishing cell lines from local tick species. The numbers of scientists and research institutes worldwide utilising tick cell lines, and the resultant outputs, are increasing exponentially. The Tick Cell Biobank has made an important contribution to this expansion, and will continue to do so. In particular, establishment of the Tick Cell Biobank Outposts in Malaysia, Kenya and Brazil will facilitate increased uptake and application of tick and other arthropod cell lines by scientists in these countries and the surrounding regions, enabling them to fully exploit the potential of these technologies in their research. In the long term, this will build and expand local and global capacity for vector-borne disease research, leading to both locally-generated and international solutions for LMIC problems.
